# Trapped in a tight spot: Scaling effects occur when, according to the action-specific account, they should not, and fail to occur when they should

**DOI:** 10.3758/s13414-017-1454-y

**Published:** 2018-01-16

**Authors:** Elizabeth S. Collier, Rebecca Lawson

**Affiliations:** Institute of Psychology Health and Society, Eleanor Rathbone Building, University of Liverpool, Bedford Street South, Liverpool, L69 7ZA UK

**Keywords:** Action, Perception, Perceptual scaling, Demand characteristics

## Abstract

The action-specific account of perception claims that what we see is perceptually scaled according to our action capacity. However, it has been argued that this account relies on an overly confirmatory research strategy—predicting the presence of, and then finding, an effect (Firestone & Scholl, 2014). A comprehensive approach should also test disconfirmatory predictions, in which no effect is expected. In two experiments, we tested one such prediction based on the action-specific account, namely that scaling effects should occur only when participants intend to act (Witt, Proffitt, & Epstein, 2005). All participants wore asymmetric gloves in which one glove was padded with extra material, so that one hand was wider than the other. Participants visually estimated the width of apertures. The action-specific account predicts that the apertures should be estimated as being narrower for the wider hand, but only when participants intend to act. We found this scaling effect when it should not have occurred (Exp. 1, for participants who did not intend to act), as well as no effect when it should have occurred (Exp. 2, for participants who intended to act but were given a cover story for the visibility and position of their hands). Thus, the cover story used in Experiment 2 eliminated the scaling effect found in Experiment 1. We suggest that the scaling effect observed in Experiment 1 likely resulted from demand characteristics associated with using a salient, unexplained manipulation (e.g., telling people which hand to use to do the task). Our results suggest that the action-specific account lacks predictive power.

Given the tight coupling between action and perception (e.g., Clark, 1999; Franchak, van der Zalm, & Adolph, 2010; Gibson, 1979/2015), the action-specific account of perception proposes that what we perceive is scaled according to our action capacity (Proffitt, 2013; Proffitt & Linkenauger, 2013; Witt, 2011, 2017; Witt, Linkenauger, & Wickens, 2016). One of the earliest findings that suggested that visual perception scales according to participants’ action capacity was that participants estimated hills as steeper after vigorous exercise than before exercising (Proffitt, Bhalla, Gossweiler, & Midgett, 1995, Exp. 5). Subsequently, Bhalla and Proffitt (1999) reported that hills were also estimated as steeper by participants who wore a heavy backpack, were elderly or in ill health, or had low physical fitness. Many later studies have reported effects consistent with the action-specific account (for reviews, see Proffitt, 2013; Proffitt & Linkenauger, 2013; Witt, 2011, 2017). Proffitt and Linkenauger (2013) suggested that perception can be scaled according to three components of action capacity: the bioenergetic cost of acting, performance variability, and action capacity pertaining to the functional morphology of the body. For example, for bioenergetics, Witt, Proffitt, and Epstein (2004) reported that distances to a target were estimated as being greater after participants had thrown a heavy ball than after they had thrown a light ball. For scaling according to performance variability, Witt and Dorsch (2009) found that goalposts were estimated as being higher by participants with worse kicking performance. For functional morphology, Linkenauger, Leyrer, Bülthoff, and Mohler (2013) used virtual reality to alter participants’ perceived hand size. They found that objects seen near the hand were estimated as being larger when the hand was rendered as smaller, and vice versa when the hand was rendered as larger. In short, the action-specific account proposes that we literally perceive the world as being scaled in terms of our ability to perform actions (for reviews, see Firestone, 2013; Linkenauger, 2015; Philbeck & Witt, 2015).

However, there are a number of concerns with the action-specific account (e.g., Collier & Lawson, 2017a, b; Durgin et al., 2009; Firestone, 2013). For example, Firestone and Scholl (2014; see also Firestone & Scholl, 2016) argued that this account relies on an overly confirmatory research strategy—that is, predicting and then finding a given effect. A comprehensive account of a phenomenon should also be able to predict the absence of an effect. Firestone and Scholl (2014) suggested employing the El Greco fallacy to test disconfirmatory predictions of the action-specific account. According to this fallacy, if perception of both the stimulus and the means of reproducing the stimulus are expected to show the same distortion following some manipulation, then the two distortions should cancel each other out, and no overall distortion should be perceived. Firestone and Scholl (2014) applied this logic to the finding that apertures were estimated as being narrower when participants held a horizontal rod that was wider than their body (Stefanucci & Geuss, 2009). For example, the participants in Stefanucci and Geuss’s second experiment estimated the width of apertures by verbally guiding the experimenter to adjust the length of a tape measure until the length matched the width of an aperture that they were told to imagine walking through. Four groups of participants were used in this experiment. The *hold* group held a long rod horizontally in front of their body, with their arms wide apart. The *hands-only* group positioned their arms in the same way as the *hold* group, but did not hold the rod. The *wear* group wore a backpack to which the rod was attached, so the rod was positioned as for the *hold* group but now participants kept their arms by their sides. Finally, the *control* group kept their arms by their sides and had no rod. The participants in the *hold* and *hands-only* groups estimated apertures as being narrower than did those in the *wear* and *control* groups. Stefanucci and Geuss interpreted this as evidence that participants who had their body widened in a functionally meaningful way perceived apertures as being less passable, and therefore narrower. Participants in Firestone and Scholl’s (2014) replication of that study either held or did not hold a rod and verbally guided the experimenter to make adjustments to visually match the width of apertures that they imagined walking through. However, instead of a tape measure, the experimenter adjusted the width of a second aperture (the *matching aperture*) that was placed perpendicular to, but was otherwise identical to, the aperture that participants imagined walking through (the *stimulus aperture*). Firestone and Scholl (2014) found that participants holding the rod estimated the apertures as being wider than did participants not holding the rod. Importantly, holding a rod should have influenced both the stimulus aperture and the matching aperture in the same way, by making them appear less passable. Thus, according to the El Greco fallacy, this should have made it impossible to detect a scaling effect, and so, although the scaling effect reported by Stefanucci and Geuss was replicated by Firestone and Scholl (2014), this in fact provided evidence against, not for, action-specific scaling.

If effects consistent with the action-specific account occur when they should not, what instead can explain their occurrence? Firestone and Scholl (2014) showed that the scaling effect on apertures that they observed disappeared if participants were given a convincing cover story for holding the rod. This suggests that the effect originally reported by Stefanucci and Geuss (2009) could have resulted from demand characteristics due to being asked to hold a rod without any explanation. Demand characteristics (Orne, 1962) can also explain other scaling effects that had originally been interpreted as supporting the action-specific account (Collier & Lawson, 2017a; Durgin et al., 2009). For example, in some of the first studies to provide evidence for the action-specific account, hills were estimated as steeper when observers wore a heavy backpack (Bhalla & Proffitt, 1999; Proffitt et al., 1995). However, Durgin et al. (2009) found that if participants were provided with a cover story for wearing the backpack, their slant estimates were no different from those of participants who did not wear the backpack. This suggests that participants who were not given a reason for the backpack manipulation may have figured out that it was intended to influence their slant estimates and adjusted their responses accordingly. Therefore, at least some scaling effects could result from demand characteristics associated with a salient, unexplained manipulation.

The reason that action-specific researchers often ask participants to imagine performing a relevant action, as in Stefanucci and Geuss (2009) and Firestone and Scholl (2014), is that scaling effects are only expected when participants intend to act (Witt, Proffitt, & Epstein, 2005). The role of intention to act in the representation of space was first investigated in electrophysiological studies on monkeys by Iriki, Tanaka, and Iwamura (1996). These authors identified neurons that fired when a raisin was placed within the monkey’s reach but did not fire when the raisin was placed beyond reach. Furthermore, after monkeys were taught to reach with a tool, these neurons adapted and now fired when raisins were placed out of arm’s reach but still within reach using the tool. However, this adaptation did not occur when the monkeys held, but never reached with, the tool (Iriki et al., 1996). This was interpreted by Witt et al. (2005) as evidence that the intention to act may be critical for changes in the representation of near space to occur.

On the basis of Iriki et al.’s (1996) findings, Witt et al. (2005) tested whether intention to act modulated the perception of near space in humans. Witt et al. (2005) found that participants estimated the distance to targets that were out of arm’s reach as being shorter after reaching to them with a tool that increased the participants’ maximum reach and made the targets reachable. However, this effect was found only for participants who actually reached with the tool. No effect was found for participants who held the tool but never reached with it. The authors interpreted this as support for their claim that action-specific effects occur only when people intend to act. Intention to act has been argued as being critical for finding scaling effects in a number of subsequent studies (e.g., Lessard, Linkenauger, & Proffitt, 2009; Linkenauger, Witt, & Proffitt, 2011; Stefanucci & Geuss, 2009; Witt & Proffitt, 2008). We therefore tested here whether scaling effects due to changes in action capacity occur if, and only if, participants intend to act.

To summarize, a comprehensive theoretical account should be able to predict both the presence and absence of an effect. Although the action-specific account has largely relied on a confirmatory research strategy (Firestone & Scholl, 2014, 2016), this account makes the disconfirmatory prediction that scaling effects should only be found when participants intend to act. In the present studies, we tested this prediction for the task of estimating aperture width.

## Experiment 1

The passability of aperture width is a good candidate for testing the claims of the action-specific account. People’s perception of whether they can walk through an aperture is dependent on their body size (Franchak, Celano, & Adolph, 2012) and can rapidly be recalibrated following an increase in body girth (Franchak & Adolph, 2014). Similarly, Ishak, Adolph, and Lin (2008) reported that people recalibrate whether their hand can fit through a variable-width aperture following an increase in hand width. Specifically, when participants wore a prosthesis on their hand that increased their hand width, they appropriately judged the minimum passable aperture width for that hand as being wider. The results of Franchak and colleagues and of Ishak et al. demonstrate that people are sensitive to changes to their action capacity following a change in the functional morphology of their body. However, crucially, these results are not relevant to the claims of the action-specific account. This account predicts that estimates of spatial properties of action-relevant stimuli should be affected by changes in functional morphology. Specifically, here the action-specific account predicts that people should perceive apertures that they intend to move their wider hand through as being narrower, but only when they intend to act in this way (Witt et al., 2005). No scaling effect on estimates of aperture size should be found if participants do not intend to act on the aperture.

In Experiment 1, in separate tasks, we tested both whether participants’ estimates of the narrowest aperture they could fit their hand through (action capacity task) *and* their estimates of aperture width (perceptual task) were affected by wearing a padded glove. The aperture apparatus, gloves, and method for measuring perceived aperture passability in the action capacity task were closely based on the methods of Ishak et al. (2008). The visual matching method used in the perceptual task was the same as that used in other work investigating the action-specific account (e.g., Collier & Lawson, 2017a, b; Linkenauger et al., 2011, b).

### Method

#### Participants

Thirty-six participants (23 females, 13 males; mean age = 21.8 years) were recruited from the University of Liverpool. All participants self-reported as right-handed and were rewarded with course credit for their participation.

#### Design

Participants were assigned to either the Intention-to-Act group or the No-Intention group. All participants completed two tasks: a perceptual task in which they estimated the width of apertures, and an action capacity task in which they judged whether they could fit their hand through apertures of different widths. These tasks are described in detail below. The Intention-to-Act group (*n* = 18) completed the action capacity task before the perceptual task, and on each trial of the perceptual task, they were asked whether they thought they could fit their hand through the aperture before estimating its width. This is a technique used by proponents of the action-specific account to ensure that participants intend to act in the way that the experimenter is interested in (e.g., Linkenauger et al., 2011, b). The No-Intention group (*n* = 18) completed the perceptual task before the action capacity task, and they were not asked whether they thought they could fit their hand through the aperture during the perceptual task. Therefore, only the Intention-to-Act group intended to act while estimating the aperture’s width.^1^

#### Stimuli, apparatus, and procedure

**Fig. 1:**
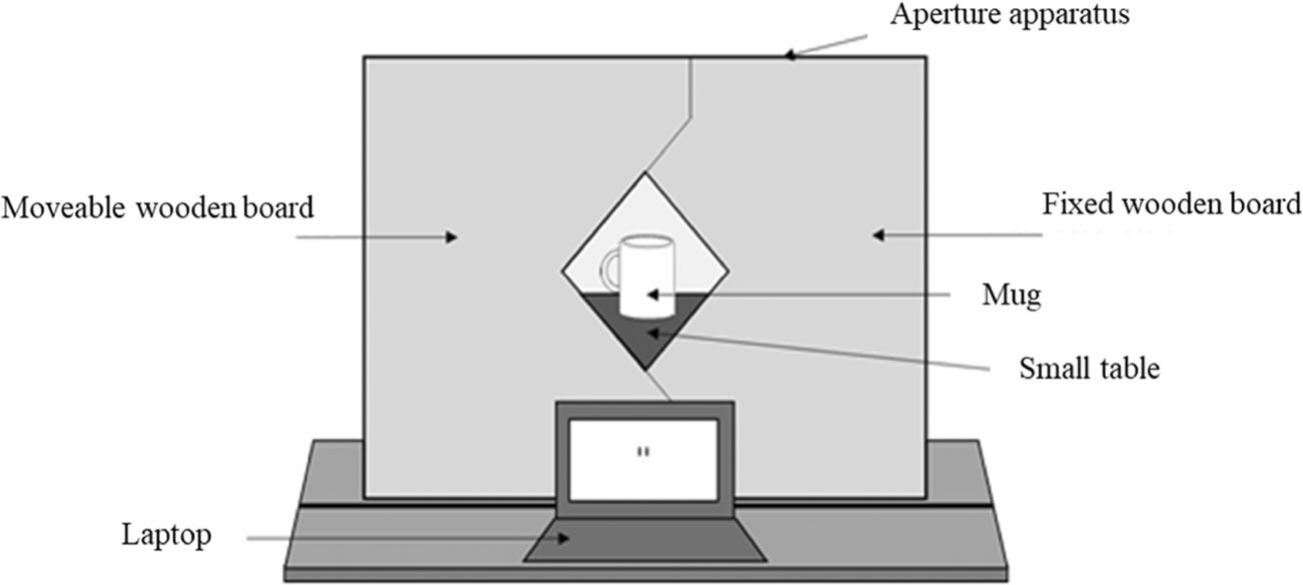
Diagram showing the aperture apparatus used in Experiment 1. The same apparatus was used in Experiment 2, except that the laptop was placed at a 90° angle to the aperture.

An aperture apparatus was created using a metal frame that held two wooden boards (see Fig. 1). One board was fixed, and the other could move to vary the width of a diamond-shaped aperture between the boards from 0 cm (minimum) to 30 cm (maximum). A mug was placed on a small table behind the aperture apparatus, with the handle facing the participant. A laptop was placed in front of the aperture apparatus, with two black lines displayed on its screen. The lines began at a default distance of 1.75 cm apart. Each press of the up arrow on the laptop keyboard moved the lines 1 mm farther apart, and each press of the down arrow moved the lines closer together by 1 mm.

Participants wore a pair of gloves throughout the experiment. The left (padded) glove had additional woolen material sewn into the little finger-side of the glove.^2^ The right (unpadded) glove had no padding. Here we refer to the hands as “padded” or “unpadded,” but the experimenter always referred to the “left” or “right” hand when communicating with participants, and participants were not informed about the padding.

##### Action capacity task

**Fig. 2:**
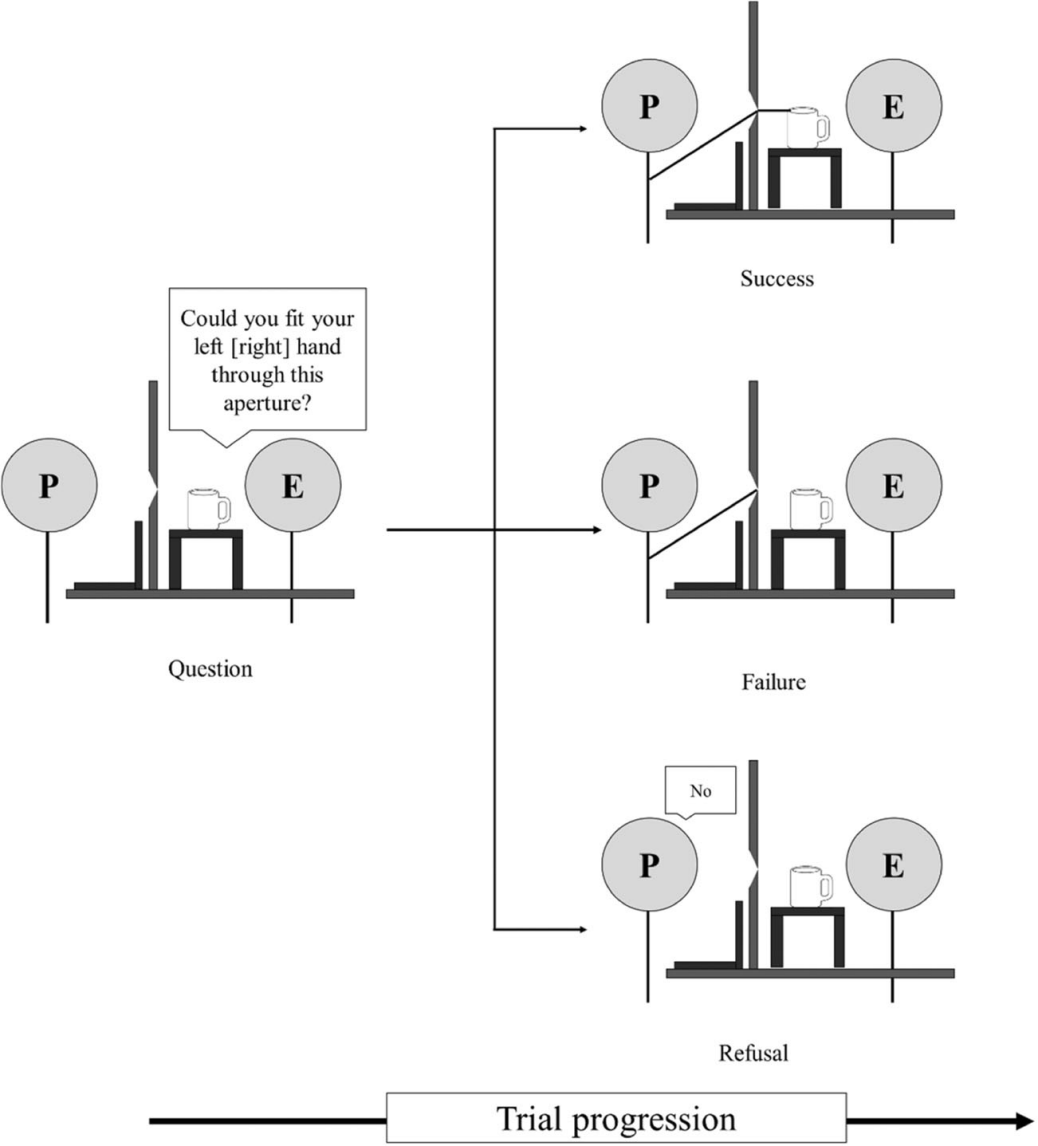
Diagram showing a participant (P) completing the action capacity task in Experiment 1. The experimenter (E) first asked the participant whether he or she could fit a hand through the aperture. The participant responded by either attempting the action (no verbal response given) or by verbally responding “no” and refusing to attempt. These responses were coded as “success” (the participant successfully reached through the aperture, top), “failure” (the participant attempted to reach through but the hand did not fit, middle), or “refusal” (the participant said that the hand would not fit through the aperture, bottom).

On every trial of the action capacity task, participants were asked whether they thought they could fit their hand through the aperture to touch the mug on the other side (see Fig. 1). If, and only if, they thought they could fit their hand though the aperture did they then attempt to actually do so. If they thought they could not fit their hand through, they verbally responded “no.” They were told to judge passability on the basis of their hand being held flat and oriented horizontally with their fingers close together and their thumb tucked under their fingers. They were told not to twist their hand, screw their fingers into a fist, or bunch their fingers together. On each trial, the experimenter told them to use either their left (padded) or their right (unpadded) hand. The responses were coded as “success” (the participant could reach through the aperture), “failure” (the participant attempted to reach through but the hand did not fit), or “refusal” (the participant said that the hand would not fit through the aperture); see Fig. 2. The aperture width ranged from 4 to 14 cm, in 0.5-cm increments. Participants judged whether they could fit their left (padded) or their right (unpadded) hand through each aperture width three times, giving 126 trials in total (2 hands × 21 aperture widths × 3 repeats), with trials presented in a different random order for each participant.

##### Perceptual task

**Fig. 3:**
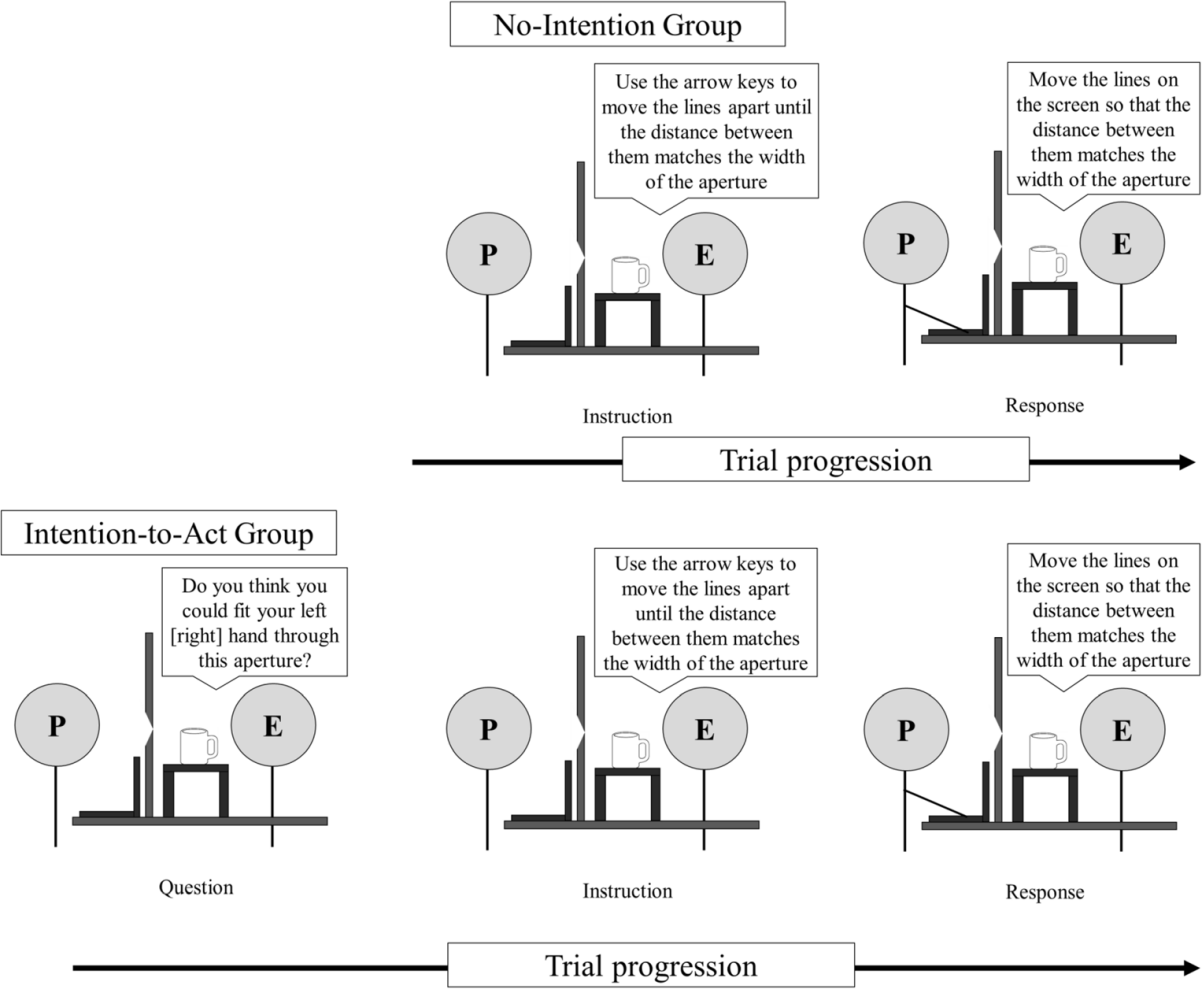
Diagram showing participants in the No-Intention group (top) and the Intention-to-Act group (bottom) completing the perceptual task in Experiment 1. For both groups, the experimenter asked the participant (P) to use the arrow keys to move the lines on the laptop screen to match the width of the aperture. In the Intention-to-Act group, the participant was also asked whether he or she could fit the left (or right) hand through the aperture, immediately before matching its width.

In this task, participants were asked to use the arrow keys on the keyboard to move the lines on the screen until the distance between them matched the width of the aperture. The participants in the No-Intention group were only told to match the width of the aperture on each trial (see Fig. 3, top). In contrast, on every trial in the perceptual task, before matching the aperture width, the participants in the Intention-to-Act group were asked whether they thought they could fit one of their hands through the aperture (see Fig. 3, bottom). Unlike in the action capacity task, here participants did not actually attempt to move their hand through the aperture.

On each trial of the perceptual task, the experimenter told participants which hand they should use to respond. For the Intention-to-Act group, this was always the same hand that the participant had just judged the aperture passability for. Participants were told to keep their other hand by their side so that it was out of sight. Between trials, they kept both hands by the sides of their body and closed their eyes until the experimenter had adjusted the width of the aperture. The aperture widths used were the same as in the action capacity task. Participants matched each aperture width once using each hand, giving a total of 42 trials (2 hands × 21 aperture widths), with trials presented in a different random order for each participant.

##### Actual aperture passability task

After participants had completed both the perceptual and action capacity tasks, the experimenter measured the actual narrowest aperture that each participant could fit his or her hands through. The experimenter opened the aperture to 15 cm and asked participants to place their hand inside it with the hand held flat and horizontally, the fingers close together, and the thumb tucked under the fingers. The experimenter then closed the aperture around the participant’s hand and asked the participant to move the hand in and out of the aperture. The experimenter adjusted the aperture until it was at the narrowest width that still allowed the participant to fit the hand through without getting it trapped. Participants were only told to move their hand during this task; they were not asked about aperture passability. The minimum passable aperture was measured for each hand, both with and without the gloves.

### Results

#### Effect of wearing gloves on actual aperture passability

To check that the glove manipulation was effective, we tested whether wearing the gloves changed the actual minimum passable aperture for each hand. We conducted a mixed analysis of variance (ANOVA), in which Hand (padded/unpadded) and Gloves (with/without) were within-participants factors, and Group (Intention-to-Act/No-Intention) was a between-participants factor. We found a significant main effect of gloves, *F*(1, 34) = 38.351, *p* < .001, *η*_p_^2^ = .53, which was modulated by a Hand × Gloves interaction, *F*(1, 34) = 40.090, *p* < .001, *η*_p_^2^ = .54. Bonferroni-corrected pairwise comparisons showed that, with gloves, the minimum passable aperture was greater for the padded hand (*M* = 10.4 cm, *SE* = 0.14 cm) than for the unpadded hand (*M* = 9.6 cm, *SE* = 0.15 cm), whereas we found no significant difference between the padded and unpadded hands without gloves (*M* = 9.8 cm, *SE* = 0.12 cm; *M* = 9.5 cm, *SE* = 0.13 cm, respectively). There were also no effect of group, *F*(1, 34) = 0.038, *p* = .8, *η*_p_^2^ = .001, and no other significant interactions: Gloves × Group, *F*(1, 34) = 1.060, *p* = .3, *η*_p_^2^ = .03; Hand × Group, *F*(1, 34) = 0.708, *p* = .4, *η*_p_^2^ = .02; Gloves × Hand × Group, *F*(1, 34) = 0.216, *p* = .6, *η*_p_^2^ = .01. Wearing a padded glove therefore significantly increased hand width, as we had intended.

#### Action capacity task: Perceived aperture passability

We tested whether participants appropriately recalibrated their perception of aperture passability to reflect the asymmetry in hand width caused by wearing the gloves. For each width tested, each hand, and each participant, we calculated the proportion of times that participants said that they could not fit their hand through that aperture in the action capacity task. Cumulative Gaussians were then fitted, from which we calculated the predicted width at which participants believed they could not fit each hand through 50% of the time (the point of subjective equality, PSE; the mean cumulative Gaussians can be found in the Appendix). These PSEs provided an estimate of the minimum aperture width that participants perceived they could fit their hand through.

PSEs were then used as the dependent variable in a mixed ANOVA in which Hand (padded/unpadded) was a within-participants factor and Group (Intention-to-Act/No-Intention) was a between-participants factor. Participants perceived the minimum passable aperture width for their padded gloved hand (*M* = 10.6 cm, *SE* = 0.16 cm) to be greater than that for their unpadded gloved hand (*M* = 9.9 cm, *SE* = 0.14 cm), *F*(1, 34) = 76.113, *p* < .001, *η*_p_^2^ = .70. We found no effect of group, *F*(1, 34) = 0.067, *p* = .8, *η*_p_^2^ = .002, or Hand × Group interaction, *F*(1, 34) = 1.579, *p* = .2, *η*_p_^2^ = .04. Thus, participants appropriately recalibrated their perception of the minimum aperture width that each gloved hand could fit through during the action capacity task, by increasing their estimates for the padded hand.

#### Perceptual task: Estimated aperture width

**Fig. 4:**
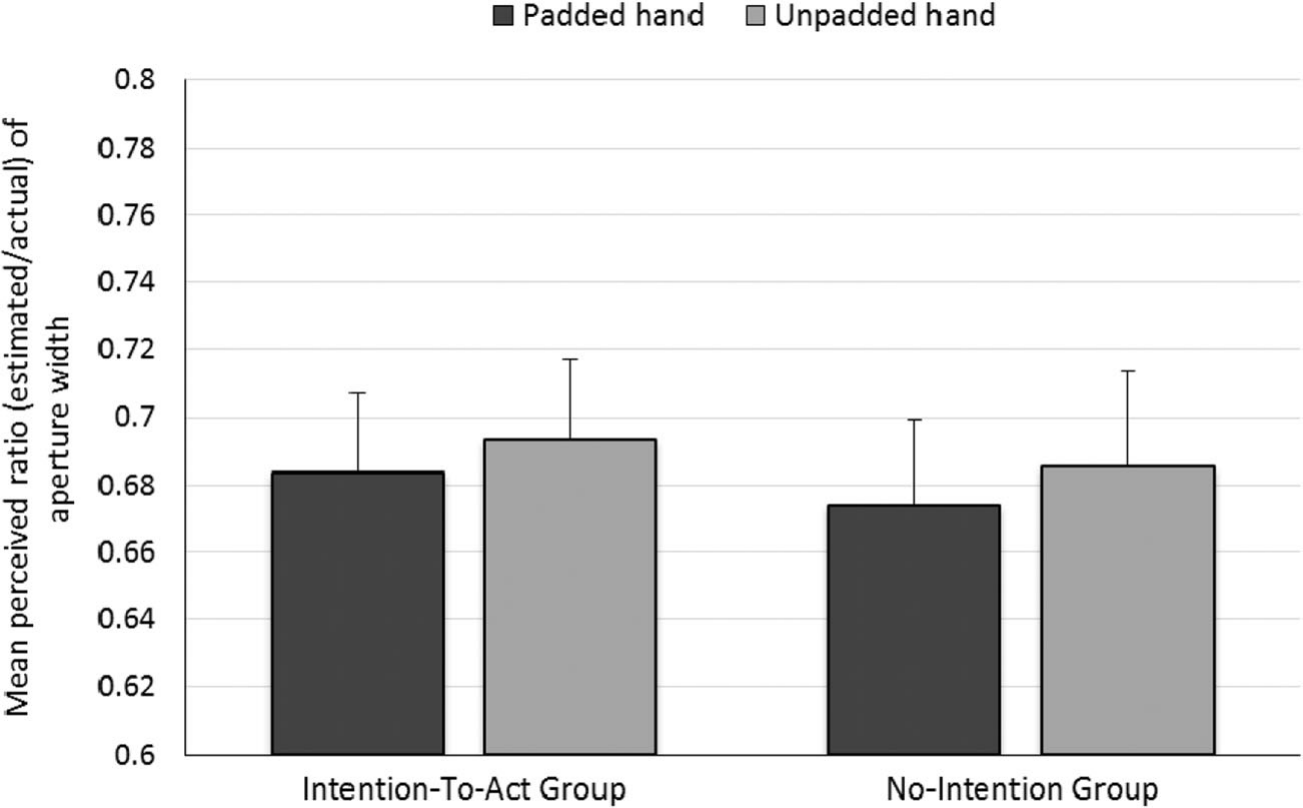
Results of the perceptual task in Experiment 1: Mean ratio of aperture size (estimated/actual) for each hand for each group. Error bars represent one standard error of the mean.

**Fig. 5:**
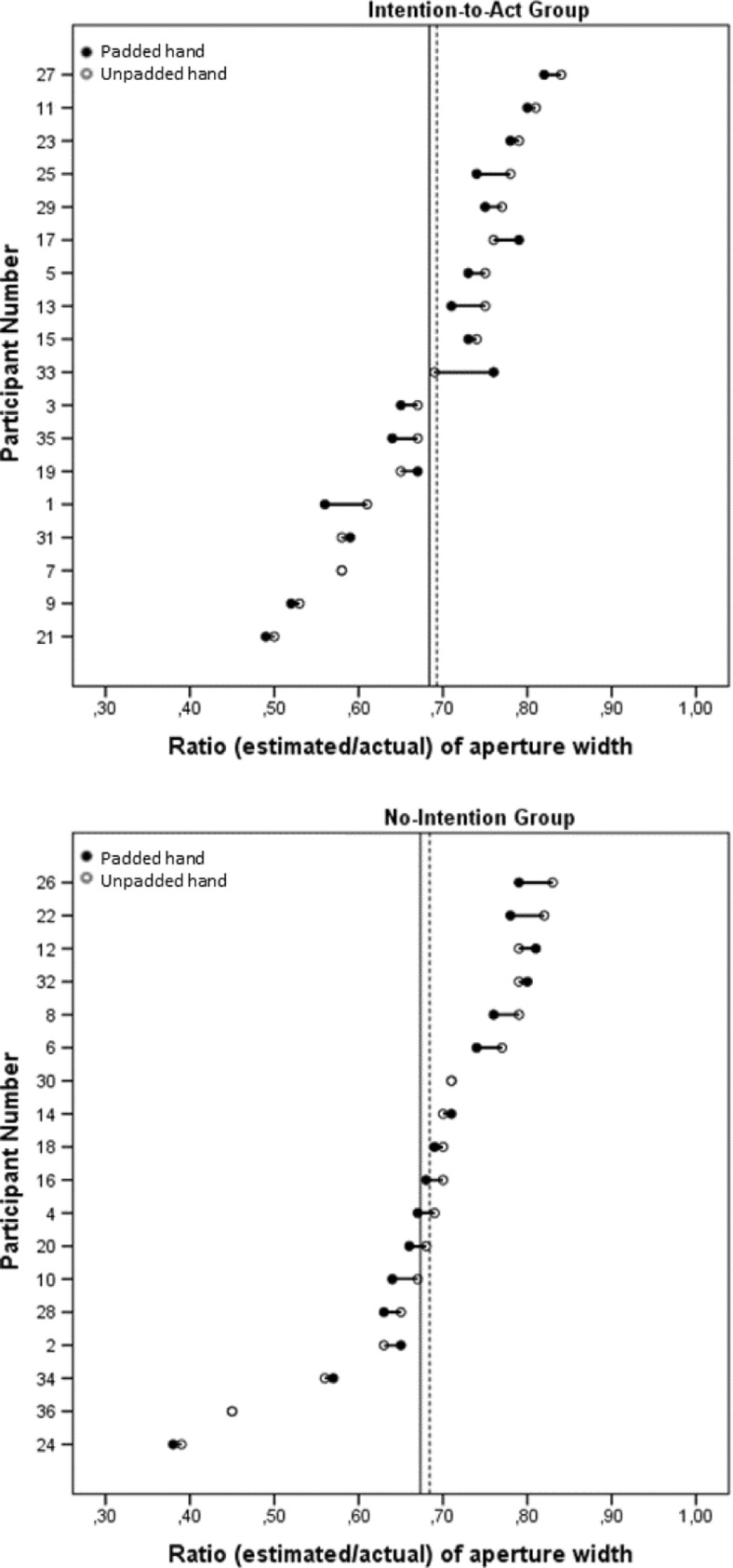
Individual estimates of aperture width (as a ratio of actual aperture width) for the padded and unpadded hands in the Intention-to-Act (top) and No-Intention (bottom) groups. The solid and dashed vertical lines show the mean ratios for the padded and unpadded hands, respectively. Participants are ordered by increasing ratio of aperture width for the unpadded hand. Cases in which only one data point is shown indicate no difference between the ratios for the padded and unpadded hands.

Finally, we tested the critical action-specific prediction that the apertures would be estimated as narrower for the padded hand by the Intention-to-Act group, but not by the No-Intention group. These ratios were calculated by dividing the estimates of aperture width by the actual aperture width and then averaging over all widths for a given hand of a participant. These ratios were used as the dependent variable in a mixed ANOVA, in which Hand (padded/unpadded) was a within-participants factor and Group (Intention-to-Act/No-Intention) was a between-participants factor. The ratios for the padded hand (*M* = 0.68, *SE* = 0.02) were significantly lower than those for the unpadded hand (*M* = 0.69, *SE* = 0.02), *F*(1, 34) = 6.557, *p* = .015, *η*_p_^2^ = .16 (see Fig. 4). Although the effect that we observed is small, this is common in the action-specific literature (see Firestone, 2013, for a discussion). There was no effect of group, *F*(1, 34) = 0.058, *p* = .8, *η*_p_^2^ = .002, nor a Hand × Group interaction, *F*(1, 34) = 0.027, *p* = .9, *η*_p_^2^ = .001. Figure 5 shows the ratios for the padded and unpadded hands given by each individual participant.

### Discussion

Padding one hand increased the minimum passable aperture for that hand. Furthermore, this change was perceived by participants: In the action capacity task, participants estimated the minimum passable aperture for their padded hand as being wider than that for their unpadded hand. The latter result is consistent with previous results (Collier & Lawson, 2017b; Ishak et al., 2008) showing that participants appropriately recalibrate their perceived action capacity following a change in the functional morphology of their hands. Of most interest theoretically was the perceptual task. Participants estimated the apertures as being narrower when they estimated for their padded as compared to their unpadded hand (see Fig. 4), but, importantly, this effect was not due only to the estimates by the Intention-to-Act group. The action-specific account claims that intention is necessary for finding the scaling effects predicted by this account (e.g., Linkenauger et al., 2011; Stefanucci & Geuss, 2009; Witt, 2017; Witt et al., 2005). Therefore, this account cannot explain the results of our perceptual task, since the participants in the No-Intention group were not asked to report aperture passability, and therefore did not intend to act.

An alternative explanation of our results is that demand characteristics could have arisen from explicitly telling participants whether to use either their left (padded) or their right (unpadded) hand to respond when they estimated the aperture width. No explanation was provided for this manipulation, and participants may have deduced that we expected to find a hand-dependent effect. As a consequence, some of the participants may, for example, have decided that they should use their visible hand as an anchor for estimating the aperture width. Since right-handers believe that their right hand is larger than their left hand (Collier & Lawson, 2017b; Linkenauger et al., 2011), this strategy could explain the results that we obtained.

## Experiment 2

The action-specific account cannot explain the results of Experiment 1, since we found a scaling effect when participants did not intend to act. Instead, it is possible that this effect arose because of the demand characteristics associated with telling participants whether to use their left or right hand on each trial of the perceptual task. Previous work has suggested that the demand characteristics associated with an unexplained manipulation can be reduced by using a cover story (Collier & Lawson, 2017b; Durgin et al., 2009; Durgin, Klein, Spiegel, Strawser, & Williams, 2012; Firestone & Scholl, 2014). Therefore, if the effects found in Experiment 1 were the result of demand characteristics, they might be eliminated by providing a cover story. In Experiment 2 we tested this possibility using a perceptual task similar to the one in Experiment 1. The participants in Experiment 2 always intended to act during the perceptual task. However, they were given a cover story for why their hand was visible near the aperture while they estimated its width. If no effect of hand padding were to occur when participants were given a cover story for the presence and locations of their hands, this would support the argument that the effects obtained in Experiment 1 were the result of demand characteristics.

We also made some changes to the experimental procedure in Experiment 2 to improve the design and to make it more consistent with previous studies in the action-specific literature. These changes included placing the laptop at 90° to the participant (as had been done in Linkenauger et al., 2011, Exp. 2). This ensured that participants could not use landmark-matching strategies while making their estimates. Also, half of the participants wore the padded glove on their left hand, whereas the other half wore it on their right hand. Finally, all participants were alerted to the difference in their hand width resulting from wearing the gloves. This was done by asking participants to squeeze their hand through a padded tube, which was hidden by a curtain, in order to reach the aperture on the other side. Since it is harder to squeeze wider hands through a tight space, we reasoned that the haptic feedback from this task would alert participants to the fact that one of their hands was wider than the other. Completing this haptic feedback phase also served to motivate our cover story manipulation in the main perceptual task. Specifically, participants were told that, as a control measure, in the perceptual task their hands should be in a position similar to the one they were in in the haptic feedback phase. The cover story did not explicitly mention the use of both the left and right hands. This was because, when using a cover story to minimize demand characteristics, it is critical that the cover story used not simply introduce a new set of demand characteristics (Proffitt, 2013) or further solidify the demand characteristics that might already exist. Thus, we opted for a cover story that explained the position and location of the hands on each trial. We reasoned that this would alleviate any demand characteristics associated with specifying which hand to use in the task, without explicitly drawing attention to the fact that both hands were being used.

In summary, in Experiment 2 we tested whether the results of Experiment 1 could be explained by demand characteristics. This was achieved by providing a cover story for why the participant’s hand was visible near the aperture while they estimated its width in the perceptual task. At the start of the experiment, participants were told that we were interested in how well they could perform basic actions while wearing thick gloves and that they would first complete a haptic task involving moving their hands through tight spaces. Then, after the haptic feedback phase and before beginning the main perceptual task, participants were given a cover story for the presence and location of their hands. We predicted that the hand padding would have no effect on the estimates of aperture width in Experiment 2, because participants would be given a cover story in the perceptual task that reduced its demand characteristics.

### Method

#### Participants

Thirty-six new participants (23 females, 13 males; mean age = 25.9 years) were recruited from the University of Liverpool. All participants self-reported as right-handed and were rewarded with course credit or a shopping voucher for their participation.

#### Design

Two new pairs of asymmetric gloves were made. In both pairs, the padded glove had 1.5 cm of foam on the little-finger-side and 0.5 cm of foam on the thumb-side, and the unpadded glove had 0.25 cm of foam on each side. The participants in the LHBigger group (*n* = 18) wore the padded glove on their left hand and the unpadded glove on their right hand, and the participants in the RHBigger group (*n* = 18) wore the padded glove on their right hand and the unpadded glove on their left.

#### Stimuli, apparatus, and procedure

All participants completed the haptic feedback phase, then the perceptual task, then the action capacity task, and finally the aperture passability task. The stimuli and setup were identical to those aspects of Experiment 1, except where described below.

##### Haptic feedback phase

**Fig. 6:**
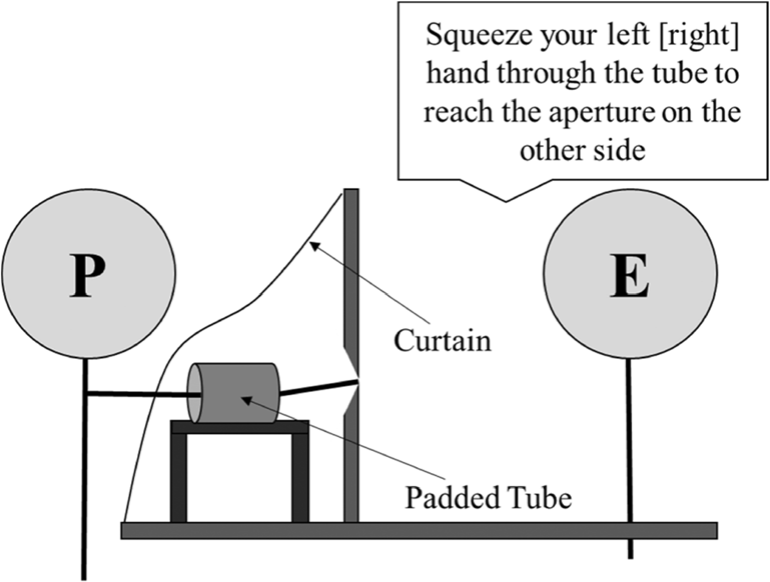
Diagram showing the setup and procedure of the haptic feedback phase in Experiment 2. The experimenter (E) has instructed the participant (P) to push a hand through the padded tube to reach the aperture on the other side.

For this task, a padded plastic tube (outer circumference = 26 cm, length = 30 cm) was placed in front of the aperture. The aperture and tube were hidden from the participant by a black curtain (see Fig. 6). Participants sat at the table and reached under the curtain to put on the gloves. They could not see that the gloves were different sizes, but we intended that participants would believe that their padded hand was wider than their unpadded hand because it was harder to squeeze their padded hand through the tube. On each trial, participants were told which of their hands they were to push through the tube to the aperture. They were told to place their thumb just inside one corner of the aperture and any other finger just inside the opposite corner, so that they could feel the horizontal width of the aperture between their thumb and finger. They then removed their hand from the tube^3^ but kept their hands under the curtain. The experimenter then adjusted the width of the aperture for the next trial. In total, participants completed 42 trials (2 hands × 21 aperture widths). The widths were the same as those used in Experiment 1 and were presented in a random order.

##### Perceptual task

**Fig. 7:**
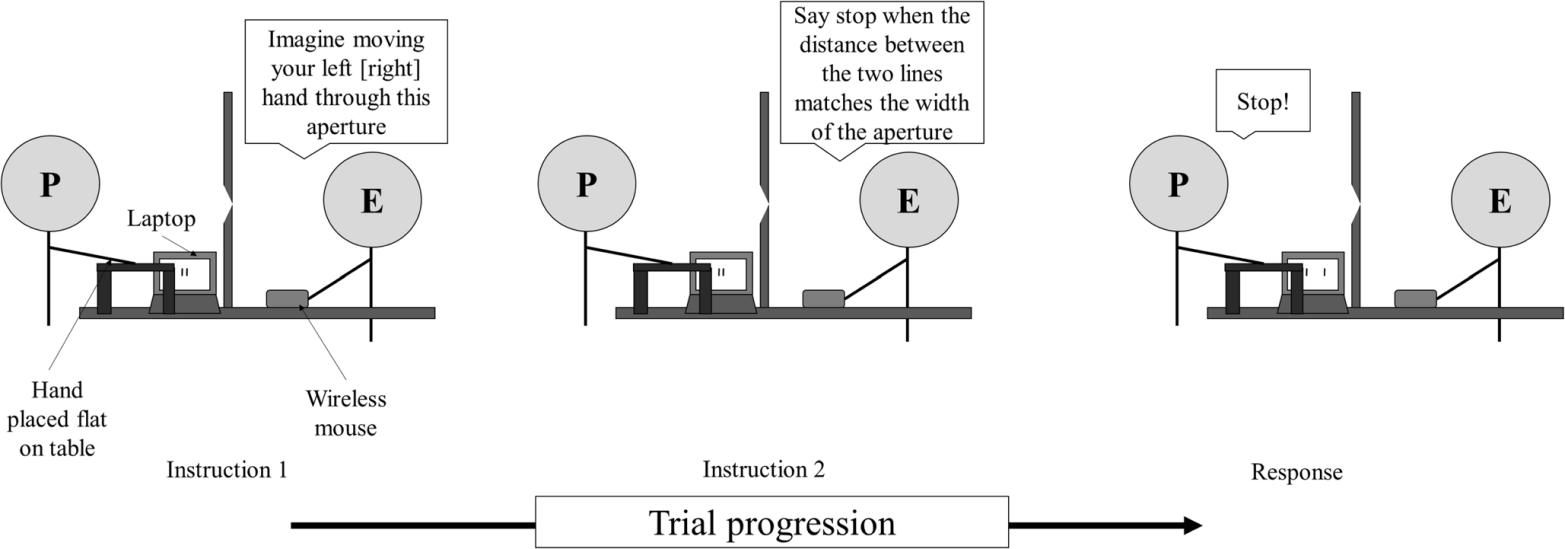
Diagram showing a participant completing the perceptual task in Experiment 2. The experimenter (E) first told the participant (P) to imagine moving the left (or right) hand through the aperture. Then the participant verbally guided the experimenter to move the lines on the laptop screen closer or farther apart until he or she thought the distance between the lines matched the width of the aperture.

This task was identical to the perceptual task used in Experiment 1, except where described below. The experimenter removed the curtain and tube apparatus used in the haptic feedback phase so that the participant could see the aperture. The same laptop that had been used in Experiment 1 was moved so that it was at 90° to the participant. On each trial, participants placed their visible hand flat on the small table in front of the aperture (see Fig. 7). Critically, participants were told that placing their hand in front of the aperture was a control measure that ensured that their hands would be in positions similar to those in the haptic feedback phase. To ensure that the participants still intended to act, on every trial they were also told to imagine moving their hand through the aperture (in the same way as in Exp. 1) as they made their width estimates. Thus, although all participants intended to act (they imagined performing the action on every trial), they were given a cover story for why they were being asked to place their hand near the aperture. Width estimates were made by verbally guiding the experimenter to move the lines on the laptop screen closer or farther apart. The experimenter used the mouse wheel of a wireless mouse to control the distance between the lines (see Fig. 7), where one click of the mouse wheel moved the lines 1 mm apart. Participants were told to say “stop” when they believed the distance between the lines matched the horizontal width of the aperture. To ensure that the estimates were as accurate as possible, participants were encouraged to request minor adjustments to the distance between the lines even after they had said “stop.” The experimenter stood behind the aperture apparatus, so she could not see the lines on the screen (see Fig. 7).

##### Action capacity task

After completing the haptic feedback and perceptual tasks, participants estimated the narrowest aperture that they thought they could fit each gloved hand through. Participants were told to imagine they were going to move their left hand through the aperture in the same way as in Experiment 1. The experimenter then opened the aperture to a width of 15 cm and slowly closed it. Participants were instructed to say “stop” when they believed the aperture was the narrowest width they could fit their left hand through. Participants were not permitted to look at their hands during the task and were asked to keep the hands by their sides.^4^ To ensure accurate estimates, the experimenter encouraged participants to request small adjustments even after they had said “stop.” The task was then repeated for the right hand.

##### Actual aperture passability task

Finally, the actual minimum passable aperture for each hand was measured, first with and then without the gloves, as in Experiment 1.

### Results

#### Effect of wearing gloves on actual aperture passability

To check that the glove manipulation was effective, we tested whether wearing the gloves changed the actual minimum passable aperture for each hand. We conducted a mixed ANOVA, in which Hand (padded/unpadded) and Gloves (with/without) were within-participants factors, and Group (LHBigger/RHBigger) was a between-participants factor. We observed a significant main effect of gloves, *F*(1, 34) = 588.183, *p* < .001, *η*_p_^2^ = .95, which was modulated by a Hand × Gloves interaction, *F*(1, 34) = 317.151, *p* < .001, *η*_p_^2^ = .90. Bonferroni-corrected pairwise comparisons showed that, with gloves, the minimum passable aperture was greater for the padded hand (*M* = 11.5 cm, *SE* = 0.11 cm) than for the unpadded hand (*M* = 10.3 cm, *SE* = 0.09 cm), whereas no significant difference was apparent between the padded and unpadded hands without gloves (*M* = 9.1 cm, *SE* = 0.12 cm; *M* = 9.1 cm, *SE* = 0.11 cm, respectively). We also found no effect of group, *F*(1, 34) = 0.004, *p* = .9, *η*_p_^2^ < .001, and no other significant interactions: Hand × Group, *F*(1, 34) = 2.967, *p* = .09, *η*_p_^2^ = .08; Gloves × Group, *F*(1, 34) = 1.029, *p* = .3, *η*_p_^2^ = .03; Hand × Gloves × Group, *F*(1, 34) = 0.912, *p* = .4, *η*_p_^2^ = .03. Wearing the padded glove therefore significantly increased hand width relative to the unpadded, gloved hand, as we had intended.

#### Action capacity task: Perceived aperture passability

We tested whether participants appropriately recalibrated their perceptions of aperture passability to reflect the asymmetry in hand width caused by wearing the gloves. The perceived minimum aperture passable by the gloved hand was calculated as in Experiment 1. This was used as the dependent variable in a mixed ANOVA in which Hand (padded/unpadded) was a within-participants factor and Group (LHBigger/RHBigger) was a between-participants factor. Participants perceived the minimum passable aperture for their padded gloved hand (*M* = 11.1 cm, *SE* = 0.19 cm) to be greater than that for their unpadded gloved hand (*M* = 10.8 cm, *SE* = 0.20 cm), *F*(1, 34) = 9.523, *p* = .005, *η*_p_^2^ = .22. Also, the perceived minimum passable aperture was greater for the RHBigger group (*M* = 11.4 cm, *SE* = 0.26 cm) than for the LHBigger group (*M* = 10.5 cm, *SE* = 0.26 cm), *F*(1, 34) = 5.912, *p* = .02, *η*_p_^2^ = .15. There was no Hand × Group interaction, *F*(1, 34) = 0.135, *p* = .7, *η*_p_^2^ = .004.

#### Perceptual task: Estimated aperture width

**Fig. 8:**
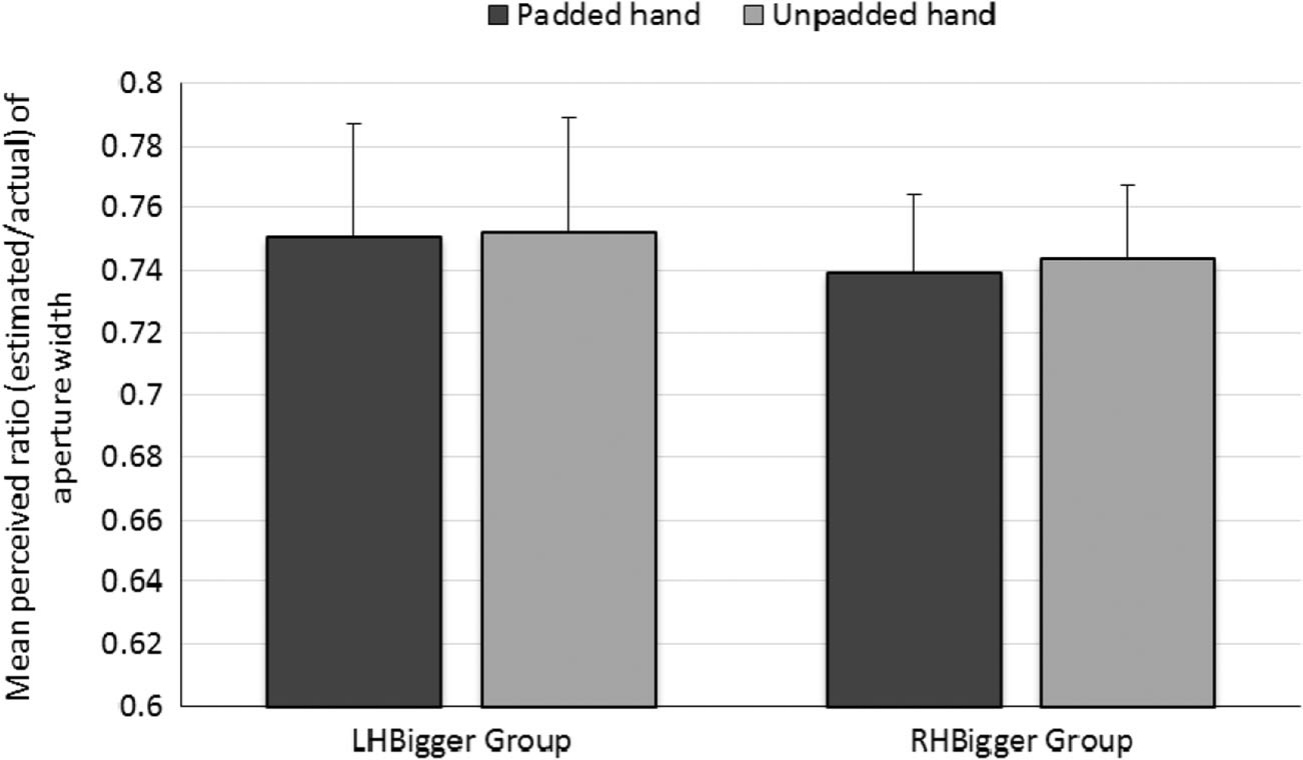
Results of the perceptual task in Experiment 2: Mean ratio of aperture size (estimated/actual) for each hand for each group. Error bars represent one standard error of the mean.

**Fig. 9:**
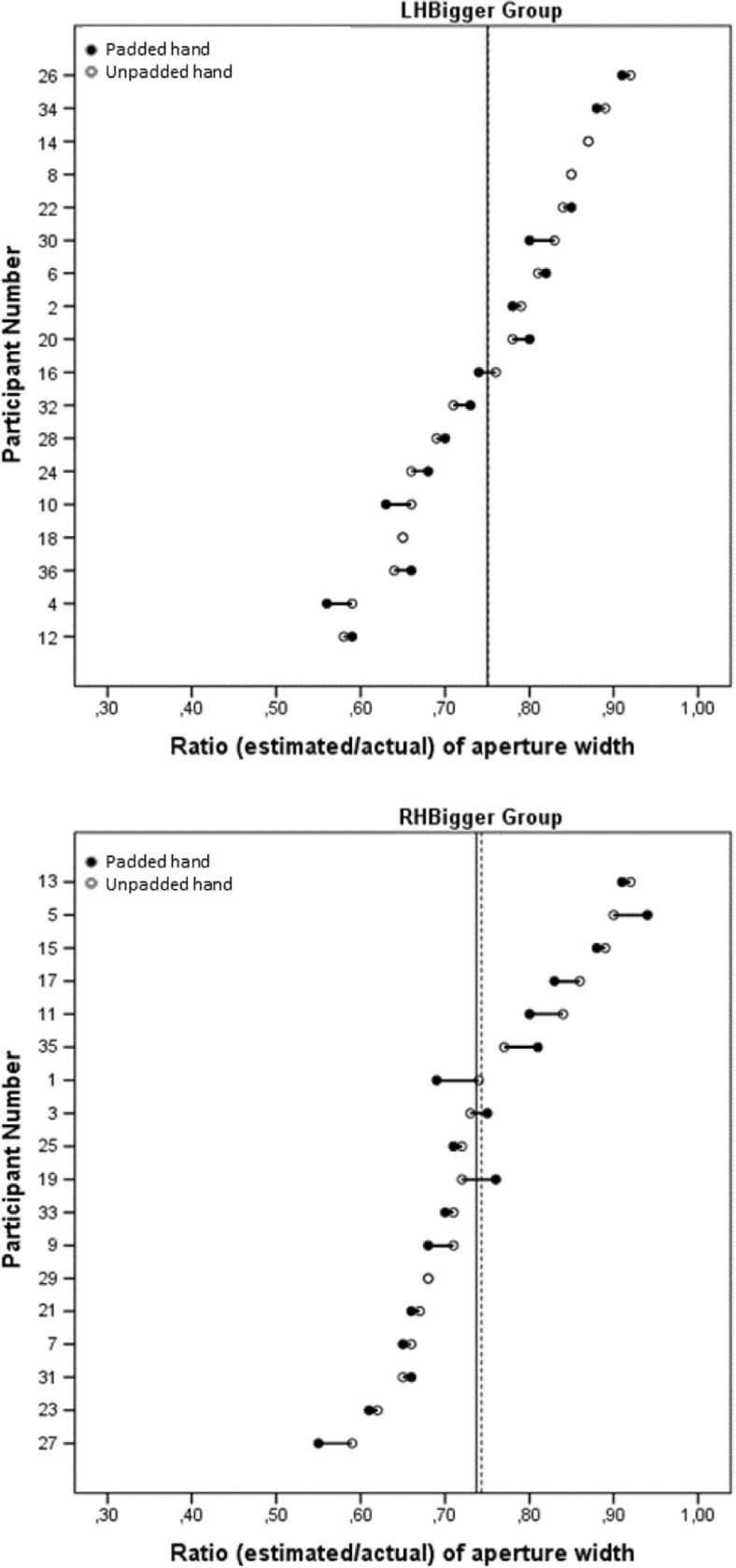
Individual estimates of aperture width (as a ratio of actual aperture width) for the padded and unpadded hands in the LHBigger (top) and RHBigger (bottom) groups. The solid and dashed vertical lines show the mean ratios for the padded and unpadded hands, respectively. Participants are ordered by increasing ratio of aperture width for the unpadded hand. Cases in which only one data point is shown indicate no difference between the ratios for the padded and unpadded hands.

Finally, we tested the critical action-specific prediction that the apertures would be estimated as being narrower for the padded hand. Ratios were calculated as in Experiment 1 and used as the dependent variable in a mixed ANOVA in which Hand (padded/unpadded) was a within-participants factor and Group (LHBigger/RHBigger) was a between-participants factor. No significant effects emerged: hand, *F*(1, 34) = 0.690, *p* = .4, *η*_p_^2^ = .02; group, *F*(1, 34) = 0.082, *p* = .8, *η*_p_^2^ = .002; Hand × Group, *F*(1, 34) = 0.180, *p* = *.*7, *η*_p_^2^ = .01; see Fig. 8. Thus, unlike the participants in Experiment 1, those in Experiment 2 did not estimate the apertures as being narrower for their padded than for their unpadded hand. Figure 9 shows the ratios for the padded and unpadded hands given by each individual participant.

### Discussion

Replicating Experiment 1, padding one hand increased the minimum passable aperture for that hand. This change was perceived by participants: In the action capacity task, the participants estimated the minimum passable aperture for their padded hand as being wider than that for their unpadded hand. Most importantly, using a cover story in the perceptual task eliminated the effect of altering action capacity on perceived aperture widths that we had found in Experiment 1. The participants in Experiment 2 were told that their hands had to be placed near the aperture in the perceptual task as a control measure to ensure that their hands were in positions similar to those in the haptic feedback phase. Our results are consistent with previous findings that have demonstrated that, even when participants intend to act, providing a cover story for a salient experimental manipulation can eliminate effects that appeared consistent with the action-specific account (Firestone & Scholl, 2014). Our present results suggest that the scaling effects found in Experiment 1 were not true perceptual changes, as proposed by the action-specific account, but were instead more likely due to demand characteristics (Orne, 1962).

It is important to emphasize that the action-specific account predicts a scaling effect in the perceptual task in Experiment 2 despite the use of a cover story. This is because, on every trial of the perceptual task, we asked participants to imagine whether they could fit their hand through the aperture before they made their width estimates. If action capacity directly influences what is perceived, as is proposed by the action-specific account, then scaling should have occurred, since we directly manipulated both the actual and perceived action capacity, and participants intended to act on every trial.

## General discussion

In the present study we were interested in biases in size perception and the role of intention to act in producing those biases. We investigated whether visual estimates of aperture width would be influenced by increases in hand size that altered action capacity. The action-specific account predicts that if a participant intends to move a wider hand through an aperture, he or she should perceive the aperture as being narrower, but that this scaling effect should not occur when participants do not intend to move their hand through the aperture (i.e., when they do not intend to act; Witt et al., 2005). However, we found that participants estimated apertures as being narrower when the width of their hand was increased by wearing a padded glove, even when they did not intend to act (Exp. 1).^5^ We then successfully eliminated this effect by providing a cover story for the presence of the hand near to the aperture, even though participants intended to act (Exp. 2). Both of these results suggest that the scaling effects that we observed were not true perceptual changes, as the action-specific account claims. Our results suggest that intention to act does not influence biases in spatial perception in the way predicted by the action-specific account. Instead, our results support previous work that has shown that the action-specific account lacks predictive power (Firestone & Scholl, 2014).

Providing a cover story can reduce the demand characteristics associated with an otherwise unexplained manipulation (Collier & Lawson, 2017b; Durgin et al., 2009; Firestone & Scholl, 2014). Bhalla and Proffitt (1999; see also Proffitt et al., 1995) reported that hills were reported as steeper when observers wore a heavy backpack. However, Durgin et al. (2009) found that if participants were told that the backpack contained equipment for monitoring their ankle muscles, their slant estimates did not differ from the estimates made by participants who did not wear a backpack. This finding suggests that participants who were not given a reason for wearing the backpack deduced that the backpack was supposed to influence their estimates of slant and changed their responses accordingly. Proponents of the action-specific account have rejected claims that their effects can be explained by demand characteristics (e.g., Linkenauger et al., 2013; Taylor-Covill & Eves, 2016; Witt & Sugovic, 2013). For example, Proffitt (2009; see also Proffitt & Linkenauger, 2013) argued that Durgin et al.’s (2009) study was not comparable to the original backpack studies because it used a 2-m ramp instead of a real hill, and the energy required to ascend such a small ramp may not be sufficient to influence perception. However, Durgin, Klein, Spiegel, Strawser, and Williams (2012) subsequently reproduced the results of Durgin et al. (2009) using a real hill, consistent with the claim that demand characteristics, rather than differences in energy requirements, produced the scaling effect on estimating hill slopes.

In Experiment 1, we found an effect consistent with the action-specific account for participants who did not intend to act. Thus, our results suggest that intention to act is not critical in producing effects consistent with the action-specific account. Intention to act has been claimed as central to obtaining the scaling effects predicted by the action-specific account. For example, Witt et al. (2005) reported that increasing participants’ maximum reach by providing them with a tool (a baton) influenced distance estimates, but only for participants who intended to reach with the tool. There is, however, an alternative interpretation of Witt et al.’s (2005) results. Franchak and Adolph (2014) showed that changes to the body are not necessarily sufficient to recalibrate perceived action capacity. They reported that pregnant women were able to accurately estimate the narrowest aperture they could walk through as this increased throughout their pregnancy. In contrast, participants who were temporarily fitted with a pregnancy prosthesis were initially inaccurate in estimating the narrowest aperture they could walk through, but after attempting the task their estimates were appropriately recalibrated. Thus, short-term changes to the body may not be sufficient to change observers’ perceived action capacity, but it can be rapidly recalibrated through acting. On the basis of this conclusion, distance estimates by participants who held—but never reached with—a tool in Experiment 3 of Witt et al. (2005) may not have been affected by holding the tool because they had not yet recalibrated their perception of their maximum reach through acting. Thus Witt et al.’s (2005) results may not have been driven by intention to act. Instead their results may have arisen because only participants who acted with the tool recalibrated their perceived reaching capacity. Note, furthermore, that this does not mean that their perception of distances changed. Instead it may only have been their *judgments* of the distances that changed because they were aware that targets were easier to reach with the tool than without it (see Firestone, 2013; Firestone & Scholl, 2016, for discussions of whether action-specific effects reflect changes in visual perception or in postperceptual judgment).

A further point is that, although intention to act is often argued to be necessary for the scaling effects predicted by the action-specific account to occur (e.g., Linkenauger et al., 2011; Witt, 2017; Witt et al., 2005), intention was not present in several studies that have been argued to support the action-specific account. For example, Bhalla and Proffitt (1999) did not mention walking up the slope to their participants, and Linkenauger et al. (2013) did not ask participants to consider or estimate the graspability of the objects they estimated the size of. Therefore, even proponents of the action-specific account are not consistent about whether intention to act is needed to induce scaling effects. Given this, one possible critique of the present work is that we focused on intention to act as a test of when scaling effects should be found and when they should not. However, countering this critique, note that the action-specific account predicts an effect for the perceptual task in Experiment 2 even if participants did not intend to act. Linkenauger and colleagues (Linkenauger et al., 2013; Linkenauger, Mohler, & Proffitt, 2011; Linkenauger, Ramenzoni, & Proffitt, 2010) have reported that just placing the participant’s hand next to an object can influence estimates of that object’s size. For example, Linkenauger et al. (2013) used virtual reality to manipulate perceived hand size. Participants were not asked to imagine grasping the object in that study, yet the authors reported that objects that appeared near the apparently larger hand were estimated as being smaller (because, according to the action-specific account, the objects were easier to grasp), and vice versa when the hand appeared to be smaller.

Another possible limitation of the present work is that, by providing a cover story in Experiment 2, we may have reduced not only the demand characteristics, but also participants’ intention to act. However, on every trial in the perceptual task, participants were told to imagine moving their hand through the aperture while making their width estimates. This manipulation has been used in studies that have been claimed to show evidence for the action-specific account based on scaling effects (e.g., Linkenauger et al., 2011; Stefanucci & Geuss, 2009). Thus, we argue that there was no less intention to act in Experiment 2 than there has been in other action-specific studies.

Our results suggest that intention to act is not critical for finding scaling effects. This is important because, if an intention to act induces scaling effects, as the action-specific account proposes, this would suggest that visual perception is cognitively penetrable (Firestone & Scholl, 2016). This, in turn, would be inconsistent with modular theories of vision, which assume that perception cannot be influenced by higher-level cognitive factors such as intention, emotion, or motivation (e.g., Firestone & Scholl, 2016; Pylyshyn, 1999). If we had found that intention to act was a driving factor in eliciting biases consistent with the action-specific account, this would challenge cognitive impenetrability and necessitate a drastic change in our understanding of how perception works (Firestone, 2013; Firestone & Scholl, 2016). Our results instead support cognitive impenetrability.

In conclusion, the results of the present study suggest that the action-specific account of perception lacks predictive power. We found a scaling effect consistent with the action-specific account when one should not have been found (Exp. 1, when participants did not intend to act), and we failed to find this scaling effect when it should have been present (Exp. 2, when participants did intend to act). In Experiment 2 we were able to eliminate effects found in Experiment 1 that appeared to be consistent with the action-specific account by using a cover story, suggesting that these effects were likely the result of demand characteristics rather than true perceptual changes. Our observers were sensitive to changes in their action capacity to act following changes in their hand size due to wearing padded gloves. However, changes in both their actual and perceived action capacities did not affect their visual spatial perception in the strong sense proposed by the action-specific account.

## Figures and Tables

**Fig. 10:**
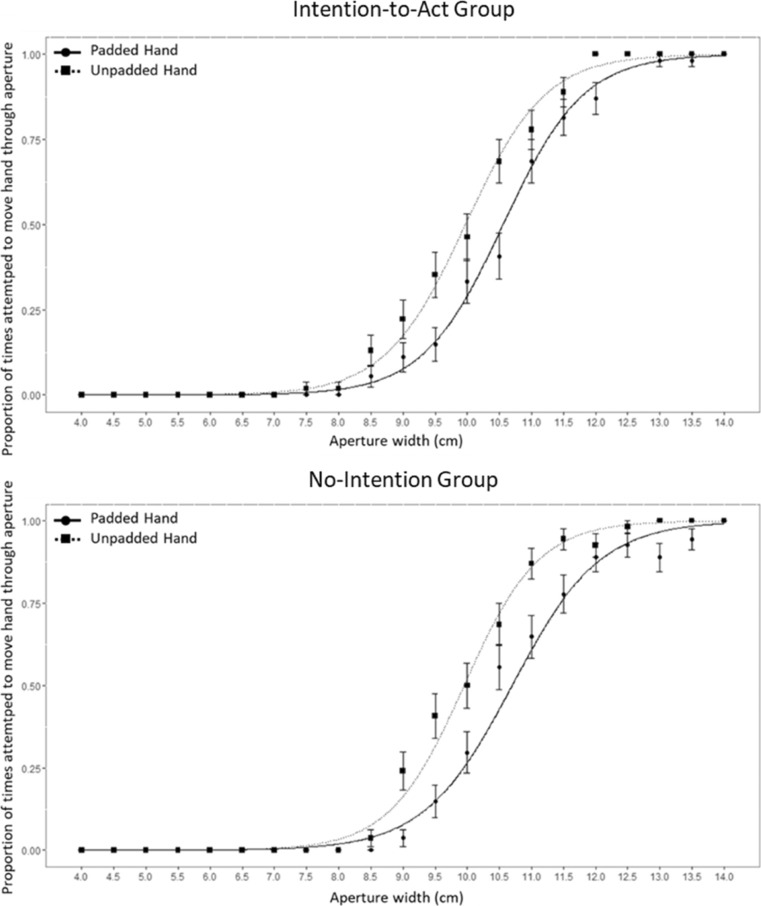
Mean cumulative Gaussian curves for the padded and unpadded hands in the Intention-to-Act (top) and No-Intention (bottom) groups in Experiment 1. Error bars show ±1 standard error of the mean.

## Footnotes

1 It is possible that at least some of the No-Intention group intended to act on the aperture, given that they were seated directly in front of it. However, this group was not explicitly asked about their action capacity, and they never acted on the aperture during the perceptual task. Thus, few of them were likely to have explicitly considered acting by moving their hand through the aperture, and any intention to act in this way in this group would likely be weak, implicit, and infrequent. In summary, the intention to put their hand through the aperture should have been stronger, explicit, and universal in the Intention-to-Act group, even if intention was not entirely absent in the No-Intention group.

2 All participants wore the padded glove on their left hand because right-handers have been shown to believe that their right hand is larger than their left (Collier & Lawson, 2017b; Linkenauger et al., 2011), so if participants were to estimate the apertures as being narrower while they wore the glove on their right hand, this could have occurred either because their judgments were affected by the change in hand size caused by the glove or because of the underlying bias in perceived hand size. Having participants always wearing the padded glove on their left hand avoided this confound. However, it introduced a second confound, namely that the same hand always wore the padded glove, so in Experiment 2 we counterbalanced which hand wore the padded glove.

3 During the haptic feedback phase, participants estimated the width of the apertures they felt between their fingers. This was done in the same way as described for the main perceptual task. These results are not reported here because the participants actually acted in this task but not in the perceptual task, making it difficult to compare the results of the two tasks. In addition, the action-specific account does not offer specific predictions for haptic spatial perception (though see Collier & Lawson, 2017b, and Linkenauger et al., 2011, for discussions of the different biases that might be expected for visual and haptic perception). The main purposes of this task were to alert participants through the haptic feedback to the difference in the widths of their padded and unpadded hands and to ensure that the cover story used in the subsequent perceptual task would be persuasive.

4 Most participants did, though, look at their hands before the task began, while the experimenter was explaining the task.

5 It might be argued that our results in Experiment 1 arose from a confound. The padded glove was always worn on the left hand, and our right-handed participants might, for example, have been less confident about their ability to pass their nondominant hand through the aperture. However, in other, similar studies (e.g., Collier & Lawson, 2017b), we have found no evidence for a baseline difference in spatial estimates depending on whether participants intended to use their dominant versus their nondominant hand. Furthermore, in Experiment 2 here this confound was removed, and there was no effect of whether our right-handed participants wore the padded glove on their left or their right hand. Instead, we suggest that the critical difference between the designs of Experiments 1 and 2 was the use of a cover story.
